# EYE-C: Eye-Contact Robust Detection and Analysis during Unconstrained Child-Therapist Interactions in the Clinical Setting of Autism Spectrum Disorders

**DOI:** 10.3390/brainsci11121555

**Published:** 2021-11-24

**Authors:** Gianpaolo Alvari, Luca Coviello, Cesare Furlanello

**Affiliations:** 1 Department of Psychology and Cognitive Sciences, University of Trento, Corso Bettini 84, 38068 Rovereto, Italy; 2 DSH Research Unit, Bruno Kessler Foundation, Via Sommarive 8, 38123 Trento, Italy; 3 University of Trento, 38122 Trento, Italy; luca.coviello@unitn.it; 4 Enogis, Via al Maso Visintainer 8, 38122 Trento, Italy; 5 HK3 Lab, Piazza Manifatture 1, 38068 Rovereto, Italy; cesare.furlanello@hk3lab.ai; 6 Orobix Life, Via Camozzi 145, 24121 Bergamo, Italy

**Keywords:** autism spectrum disorders, behavior imaging, computational phenotyping, eye contact, heterogeneity, preschool children

## Abstract

The high level of heterogeneity in Autism Spectrum Disorder (ASD) and the lack of systematic measurements complicate predicting outcomes of early intervention and the identification of better-tailored treatment programs. Computational phenotyping may assist therapists in monitoring child behavior through quantitative measures and personalizing the intervention based on individual characteristics; still, real-world behavioral analysis is an ongoing challenge. For this purpose, we designed EYE-C, a system based on OpenPose and Gaze360 for fine-grained analysis of eye-contact episodes in unconstrained therapist-child interactions via a single video camera. The model was validated on video data varying in resolution and setting, achieving promising performance. We further tested EYE-C on a clinical sample of 62 preschoolers with ASD for spectrum stratification based on eye-contact features and age. By unsupervised clustering, three distinct sub-groups were identified, differentiated by eye-contact dynamics and a specific clinical phenotype. Overall, this study highlights the potential of Artificial Intelligence in categorizing atypical behavior and providing translational solutions that might assist clinical practice.

## 1. Introduction

Shared frameworks or systematic behavioral indicators to analyze and evaluate the intervention of children with autism conditions are still lacking in clinical practice. A major cause of this is the high heterogeneity of the spectrum, both in developmental trajectories and in response to treatment [1,2,3,4,5,6]. This wide variability recalls the need to adapt the intervention to individual child characteristics; however, there is a shortage of effective behavioral markers to identify better-tailored programs [7,8]. Some factors have been considered in the literature as moderators, but the results are mixed, and their specific importance still needs to be investigated [7]. Possible concerns may arise from using behavioral correlates designated for clinical diagnosis that may not be sensitive enough to measure subtle differences in either improvement or sub-group differentiation [9]. The research effort should be directed towards structuring systematic methodologies based on fine-grained descriptors that are suitable to measure more specific behavioral variables. Such systems could be helpful to tackle spectrum stratification more systematically and to provide additional information. In this study, we aim to explore the application of an Artificial Intelligence (AI) framework based on combining video processing models and machine learning analytics to expose fine-grained behavioral metrics that may help address this challenge in unstructured settings. In particular, we examined eye-contact features through a computational phenotyping approach and employed unsupervised clustering to explore the categorization for the spectrum.

Behavior imaging involves computational sensing and modeling techniques to analyze human behavior through images and has proven great potential in clinical domains [10,11,12,13]. These approaches may provide tools that assist clinicians to monitor behaviors and structure personalized interventions through refined, systematic indicators [14,15,16]. However, the application of AI-based systems on the intervention of autism conditions is still underdeveloped and at an experimental step. The primary difficulty with real-world data from intervention sessions is that analysis is complex and needs a lot of versatility. Within the context of autism, it is well-known that it is crucial to assess behavior in settings that are as naturalistic as possible [1]. Maintaining a non-invasive approach is essential to prevent affecting the therapist-child interplay. In addition, fine-grained analyses have to deal with the high level of the dynamics of the setting, in which both child and adult are constantly moving and which includes periods of interrupted or low-quality signals. For these reasons, the application of AI modeling in the intervention scenario is usually based on restructuring interaction and setting, which often compromises the applicative value [17,18].

The more the interaction and the environment are structured, the better the quality of the data collected; however, this comes at the high cost of limited flexibility [19]. In most studies, the trade-off has been weighted in favor of more efficient model performances, resulting in a lack of translational solutions [18]. Research needs to move towards designing more balanced computational methods that account for data quality yet emphasize the ecology of interactions. Thus, it will be feasible to deliver effective AI-based systems that can be scaled to real-world scenarios and provide support for clinicians and therapists working across the autism spectrum. The novel contribution of this study in this field is the implementation of a complete system for eye contact analysis and its validation in real clinical environments.

### 1.1. Gaze Patterns

Lack of eye contact is an iconic trait on the autism spectrum [20,21]. Maintenance of sustained eye contact may significantly enhance the quality of the social experience as well as increase the likelihood of success in responding properly to stimuli and prompts, in addition to potentially improving the acquisition of adaptive social competencies [21,22,23]. As already pointed out, children with autism, even at an early age, show marked difficulties in gaze integration and an atypical response to adult gaze [24,25,26,27,28]. Therefore, gaze integration is already a prominent goal in early intervention programs [21]. Learning appropriate gaze modulation early in social interaction may enhance success in many domains and potentially improve intervention outcomes of young children with ASD [29,30].

In this research area, AI has found many applications in both symptom monitoring [11,31] and treatment, especially through robot-mediated therapy [16,32,33]. Notably, computational approaches introduce the possibility of collecting quantitative and fine-grained measures with high temporal sensitivity. Most of these approaches were designed upon employing advanced eye-tracking technologies through wearable devices (i.e., smart-glasses) [34,35,36], frontal cameras [32,37,38,39,40,41] or strong interaction structuring [11,37]. The major drawback of implementing these techniques remains the constraint of operating in not-so-naturalistic environments [17,18]. Despite the advances and the considerable appeal of this area of study, there is still a lack of examples for eye contact detection with sufficiently ecological methodologies in autism research. Given the relevance of integrating gaze into intervention programs, this issue is an important goal.

Additionally, examples of the role of gaze patterns in defining different shadings of the spectrum are also lacking in terms of a functional perspective. Most studies focused on discriminating between those diagnosed with ASD and typically developing peers [30,34,42]. To the best of our knowledge, very few studies have investigated the role of gaze in stratifying the autism spectrum. Campbell and colleagues [43] investigated the role of variability in attention to direct gaze in differentiating the autism spectrum. They employed unsupervised clustering on 20-month-old toddlers with ASD based on visual response to dyadic stimuli from videos. The analysis identified three different sub-groups that were compared for verbal, social, and adaptive functioning skills [43]. The cluster that exhibited limited attention to social scenes subsequently demonstrated a poor outcome at 3 years of age; conversely, the sub-group with good attentional abilities developed verbal abilities and is high functioning. The results of this work confirm that gaze analysis may have an interesting clinical role, both in addressing spectrum heterogeneity and as a predictor of outcome [43]. In a different approach, Fabiano and colleagues [30] used a combination of hand-crafted and raw gaze variables with demographic characteristics [44,45], such as age and gender, to classify multiple levels of ASD risk. Features included the location and duration of gaze fixations measured by eye-tracking in children between 6 and 132 months of age. They employed several classifiers (random forest, decision tree, deep feedforward neural network), showing that the different classes (low, medium, high, and ASD) correspond to different patterns that can be used to classify risk. The results confirmed the potential of gaze as an indicator that needs to be further explored by investigating the presence of sub-groups within children with ASD. In addition, the analyses showed that age is an important factor in classifying ASD risk, resulting in an overall accuracy of 93.45% [30]. More recently, Latrèche and colleagues [46] investigated the role of social orientation in modulating treatment outcomes in preschool children with ASD. They employed eye-tracking technologies to measure subjects’ attentional patterns while watching videos of an adult engaging in child-directed speech. The results confirmed that the degree of attention to the adult’s face strongly correlated with the severity of autistic symptoms at baseline and also predicted improvement after treatment. Children with ASD who stared less at the actress’s face and avoided eye contact suffered more impairment in the socio-communicative domain and showed less after-treatment improvement, particularly in the verbal domain [46].

Overall, the results are promising and highlight the importance of social-attentive skills in categorizing the autism spectrum and for predicting treatment outcomes [30,43,46]. Gazing may be a relevant feature, but further investigations and less intrusive methodologies are needed. Behavior Imaging approaches may be suitable to address this need, offering interesting alternatives for the ecological measurement of children’s behavior through video, which can be implemented in systems with applicative value in clinical practice [18].

### 1.2. Current Study

This work is focused on developing an AI-based method for the ecological analysis of therapist-child interactions through video capable of extracting dyadic gaze coordination episodes. The purpose of this study is to test the validity of computational solutions to systematically analyze the socio-attentional components of the interactions and identify behavioral indicators that may allow for the identification of sub-groups within the spectrum.

We considered eye contact coding for analysis because it is a major impairment in autism and because of its crucial role in the early intervention [29,46]. We collected video recordings (around 60 min each) of ADOS administration sessions of children with ASD in preschool age. We included videos at different resolutions, from low to high, and in different rooms of the same laboratory, from small to large, to design a more resilient and translational framework.

For gaze analysis, we developed a combined AI-based approach based on a module to extract multi-person body and head pose keypoints [47] and a module to derive a 3D vector of gaze direction frame-by-frame from wild videos [48]. We further developed a system for derivation of eye contact periods experienced between therapist and child during unconstrained interactions. The model was validated by matching the output with hand-coded features. Continuous interactive sequences of about 10 min were extracted from five different videos (with different video resolutions and in different lab rooms) for a total of more than 70,000 frames. The sequences were hand-coded frame-by-frame, and the results were compared with the model’s output to evaluate the performance and understand under which conditions the data quality stayed too low.

Finally, we tested the gaze features for autism spectrum stratification based on machine learning methods. We hypothesized that based on our metrics, it would be possible to identify sub-groups with different levels of functioning and symptom severity through unsupervised clustering and validation on clinical variables.

The perspective of this exploratory study is to contribute by emphasizing the role that computational solutions can play in identifying systematic and ecological approaches to categorize atypical child behavior. Our method was developed by enhancing systems that are suitable for analysis in real-world scenarios [47,48]. Identifying systematic and refined behavioral indicators that categorize the heterogeneity of the autism spectrum and that are predictive of treatment outcomes can be used to help clinicians monitor and design better-tailored interventions.

## 2. Materials and Methods

### 2.1. Data Collection

All analyses and data collection were carried out in accordance with the ethical standards of the Italian Association of Psychology (AIP) and the Ethics Committee of the APSS (Trento, Italy). The study involved 85 (11F, 74M) preschool children (<6 years of age) with a confirmed diagnosis of Autism Spectrum Disorders (ASD). All the participants were Italian and recruited within ODFLab patients. All families involved in this study were well informed about the procedure and agreed to written informed consent. They also were aware of the possibility of abandoning the procedure at any time.

The diagnosis of ASD was confirmed through a comprehensive assessment of the children’s functional profile and validated through a clinical judgment by an independent clinician based on DSM-V criteria [49] and through the administration of the ADOS-2 [50]. Population characteristics are summarized in Table 1.

Inclusion criteria required that the subjects had a diagnosis of ASD and that they had been assessed within 6 years of age. During the clinical evaluation, psychological tests were administered to assess general cognitive functioning and social skills. All assessment meetings were video-recorded. In particular, videos of the ADOS-2 administration sessions were collected. Clinical variables collected in the study included ADOS-2 raw scores for social abilities and symptom severity and the Griffiths Developmental Scales (GMDS-ER) for an overall assessment of cognitive development quotient and related subscales for all the participants.

The ADOS-2 is the golden standard for the diagnosis of autism and is carried out by an experienced trained specialist. The administration procedure consists of a sustained semi-structured play interaction between the clinician and the child to elicit different socio-cognitive skills. The instrument is structured in 4 different modules according to the child’s chronological age and level of expressive language. Each module is divided into social abilities (SA) and repetitive and restricted behaviors (RRB) subscales, combined into an overall comparison score to classify the severity of the child’s autistic symptoms. In the present study, the raw scores have been included; as for the toddler module (suitable for younger children), it is not possible to compute a standardized score [50].

The GMDS-ER are developmental scales (also normalized in an Italian sample) administered to children in a laboratory setting through semi-structured activities to assess different domains of mental development in young children. The testing provides a global developmental quotient (GQ) and specific scoring on six different subscales of cognitive functioning, including gross motor, hand-eye coordination, communication, social, performance, and practical reasoning abilities. In this study, the GQ and the subscale scores were considered; the practical reasoning scale was excluded because it is not administered to young children [51].

### 2.2. Videos Specifics

The video recordings considered for the attentional pattern analysis included play interactions between therapists and children during the administration of the ADOS-2. The average duration of the recordings was approximately 1 h, but it varied with the child’s responsiveness and the quality of the interplay (duration M = 63.91 min, SD = 26.2). The play activity with the child is kept spontaneous by the therapist, although using standardized materials and a predefined sequence.

The videos were all recorded in the same laboratory, the ODFLab, but in four different rooms, two of which were larger (around 28 m^2^) and two of which were smaller (around 12 m^2^). Acquisition of the recording was carried out by using a single environmental camera in the corner of the room. The location and resolution of the cameras varied based on the room, which ranged across 384/640/720/1280/1920 pixels of width. Videos at 384 px were recorded with a Canon VC C4 camera, videos at 640 px with a Canon VB C50i camera, videos at 720 px with an AXIS 213 PTZ network camera, and videos at 1280/1920 px with a PTZOptics PT12x SDI WH G2 camera. The video cameras employed were not additionally calibrated. Details regarding video resolution in the sample are shown in Table 2.

The resulting data collection were recordings with a high variability of the content, both in terms of the interaction and the video quality of the material. The data can vary from high-resolution videos shot in a relatively small room to low-resolution videos taken in a larger room, where the subjects were more distant and less clearly visible. Such variability represents both a drawback and a resource. While it complicates testing and weakens video analysis performance, it also requires the design of a system that is more resilient to data variations.

The study’s primary objective was to define a resilient framework to extract attentional patterns from our real-world clinical data automatically. Details about the model development are described in Section 2.4.

### 2.3. Related Work

The application of AI-based models for the analysis of attentional patterns has recently advanced, with promising results [11]. The potential again relies on the opportunity to automatically measure attentional behavior through video and extract quantitative parameters in a systematic way. However, predicting gaze direction in real-world scenarios has been proven challenging. The strong variability of the environment, the occlusion of the image, and the dynamism of the interaction remain difficult variables to manage.

Most systems have been integrated to analyze gaze with a frontal camera through eye recognition and geometrical segmentation [52,53]. However effective, the strong limitation of these approaches is that they are based on heavy interaction structuring and rely on a fixed light source. They are not suitable for unconstrained environments, nor for analyzing dynamic interactions within the clinical setting [48].

An alternative is appearance-based methods that learn more direct gaze mapping using large annotated datasets [54]. These methods for gaze estimation work well in everyday settings, yet most of the state-of-the-art models are still being developed and evaluated based on datasets collected under controlled conditions in the laboratory, often acquired with a frontal camera. These conditions are constrained by limited variability in appearance and little change in head pose [54,55,56,57].

Regarding the specific analysis of eye contact episodes in dynamic interactions, there are no benchmark designs. This is because eye contact recognition does not require only an accurate estimation of gaze direction and information about the position and orientation of the target. A few examples attempted to address this issue by offering advanced solutions also using standard cameras in literature [58]. Smith and colleagues [59] employed a classification approach to determine eye contact from a camera video. Yet, their methodology required a priori knowledge about the size and pose of the target [59]. Similarly, Parekh and colleagues [60] developed a Convolutional Neural Network (CNN) architecture that recognized eye contact. Their method performed well; however, it required the subject to be stationary in front of a camera [60]. Müller and colleagues [58] developed a novel approach to recognizing eye contact in multi-person interactions to address this issue. The setting consisted of a setup of 8 different environmental cameras placed around 4 adults intruding while sitting. The model combined both gaze direction information and speech (determined by analysis of facial action units), assuming that people tend to look at the person who is talking during conversations [58,61]. The model was further evaluated on datasets of natural group interactions and performed better against more standard approaches [57,58].

Interesting examples are also available in the context of multi-person interactions [58,60]. Although efficient, these solutions stay constrained to highly structured environments and are not suitable for naturalistic clinical settings, where children and therapists often rapidly change both position and orientation. Designing a system suitable for dynamic interactions and real-world scenarios is the primary goal of the present study.

### 2.4. Model Design

We aimed to develop a complete eye contact detection system (EYE-C) well suited to analyze collected clinical videos. The first objective was to implement a computational solution for extracting multi-person gaze directions in naturalistic videos. To address this problem, we designed a system based on state-of-the-art pre-trained algorithms composed of (1) a module for extracting the head position of targets in the image [47] and (2) a module for estimating a frame-by-frame gaze direction vector [48].

For the (1) step, we used OpenPose, which is a CV model that can do real-time multi-person 2D pose estimation from in-the-wild videos [47]. The model takes as input the colored image and produces the 2D coordinates of the anatomical keypoints for each person in the image. The OpenPose pipeline consists of a first step in which the input RGB image is fed to a multi-stage CNN architecture, initialized with the VGG-19 model, and then fine-tuned [62]. In the first set of stages, a feedforward network predicts the 2D confidence map of the body keypoints. In the second stage, Part Affinity Fields (PAFs) are predicted, representing a degree of association between the keypoints and enabling body parts to integrate into a full-body pose [47,63]. In the end, the confidence map and PAFs are parsed through inference to produce 2D keypoints of all people in the image [47]. The model was evaluated on multiple datasets [64,65] and compared against Mask R-CNN [66] and AlphaPose [67], achieving the best performance considering the trade-off between speed and accuracy in the COCO Challenge 2017. The output of the model consists of a JSON file of 135 landmarks of different body parts divided into 3 blocks: body + foot, hand, and face detection.

We employed OpenPose to extract the features from the first main block (body + foot) and then computed the head bounding boxes of the targets by using the keypoints of ears, eyes, nose, and neck. Once we extracted the therapist and child’s head coordinates in the video frames, we can apply the gaze estimation module.

In the second module, (2) we used Gaze360, an appearance-based model capable of extracting a 3D gaze vector from 2D videos in-the-wild [48]. Given the absence of real-world datasets to estimate gaze, the authors first collected a large-scale dataset for gaze-tracking in unconstrained images. The dataset is the largest publicly available dataset and consists of 238 subjects in both indoor and outdoor environments with labels of 3D gaze coordinates in many head poses and distances [48]. Based on the dataset, the authors further implemented a model for gaze direction estimation. The architecture of Gaze360 is based on bidirectional Long Short-Term Memory (LSTM) capsules, which provide an average of the modeling sequences in which the output depends on both previous and future inputs [48]. Thus, a window of 7 consecutive frames of head crops is used as input (centered around the target frame) to predict gaze. In the first stage, the head crop of each frame is processed individually through a CNN, which produces 256-dimensional features. In the second step, the features are fed to the bidirectional LSTMs to produce compact representation vectors. Finally, vectors are concatenated into fully connected layers to predict both 3D gaze coordinates and a quantile error estimate [48]. The architecture was evaluated cross-dataset using several benchmark datasets of high- and low-resolution 3D gaze [48,56,59,68]. The model was further fine-tuned into new domains using a self-supervised approach and improved performance across all datasets. The large variability of the Gaze360 dataset and the cross-domain adaptation of the model allowed for excellent performance even in unseen videos from uncurated online media sources, such as Youtube videos, demonstrating flexibility and robustness [48]. The final output of the model is represented by a coordinate matrix of the gaze vector g for any head crop in each frame of the video. The coordinates in Gaze360 are computed in a spherical system and expressed in observing the camera’s Cartesian perspective system g = (x,y,z). The origin of the vector represents the center of the head (based on the coordinates of the eyes, mouth, and nose) and the coordinates (expressed between 1 and −1) define its direction. For example, if g = (0,0,−1)**,** the target is looking directly at the camera, regardless of its position. In this manner, the estimation of gaze vectors is based only on head crop’s appearance and without any other global information from the environment [48].

In summary, in our study we first combined the two modules using (1) OpenPose to extract head crops and then fed them to (2) Gaze360 to compute therapist and child gaze vectors in our dataset. We rendered all clinical videos by drawing the headboxes and gaze vectors to double-check the result. From video inspection, it was evident that the model performance dropped during periods of high interaction dynamism, i.e., when the child moved around the room and frequently changed distance and head orientation relative to the camera. In these cases, the head recognition module failed, producing head boxes that were generally smaller and varied a lot in size during short sequences. The results of Gaze360 are based upon the information of multiple consecutive frames [48]. Thus, headboxes that vary a lot in size over a few seconds compromised gaze direction estimation, often resulting in faulty vector predictions. In addition, this effect was more noticeable in videos recorded in larger rooms, where the distance to the camera was higher and the headboxes were smaller. Overall, the performance suffered heavily in the most dynamic periods of the session, both for the size variability and the reduced dimension of the head crops.

We proceeded in two directions to try to solve these problems. To better handle larger settings, (i) we increased the size of the headboxes by 50%, providing bigger input images for gaze estimation. In addition, to cope with moments of high mobility of the subjects, we (ii) forced a matched dimension of the headboxes for continuous sequences from a single target, normalizing the shape of the head crops according to the largest size recorded in short consecutive frame sequences. In practice, we increased the overall size of the head crops fed to the gaze estimation module and we normalized the headbox shape in consecutive frames, keeping the headbox constant in video sequences to manage the headbox variability in dynamic contexts. Model validation is discussed in more detail in Section 2.4.1 and Section 2.4.2.

#### 2.4.1. Eye-Contact Detection

Once the 3D gaze coordinates of the child with ASD and the therapist were extracted, the challenge was to successfully build a function to extract the periods of eye contact between targets in wild 2D clinical videos. The system needed to be resistant to variance in predictions and flexible to different setting conditions to accomplish this task. Eye contact periods were defined based on the relationship between therapist and child frame-by-frame gaze estimations. One subject was looking at the other if the gaze vector was directed toward the other’s head. If both subjects were looking at each other for a certain amount of time, then eye contact was present.

**Figure 1:**
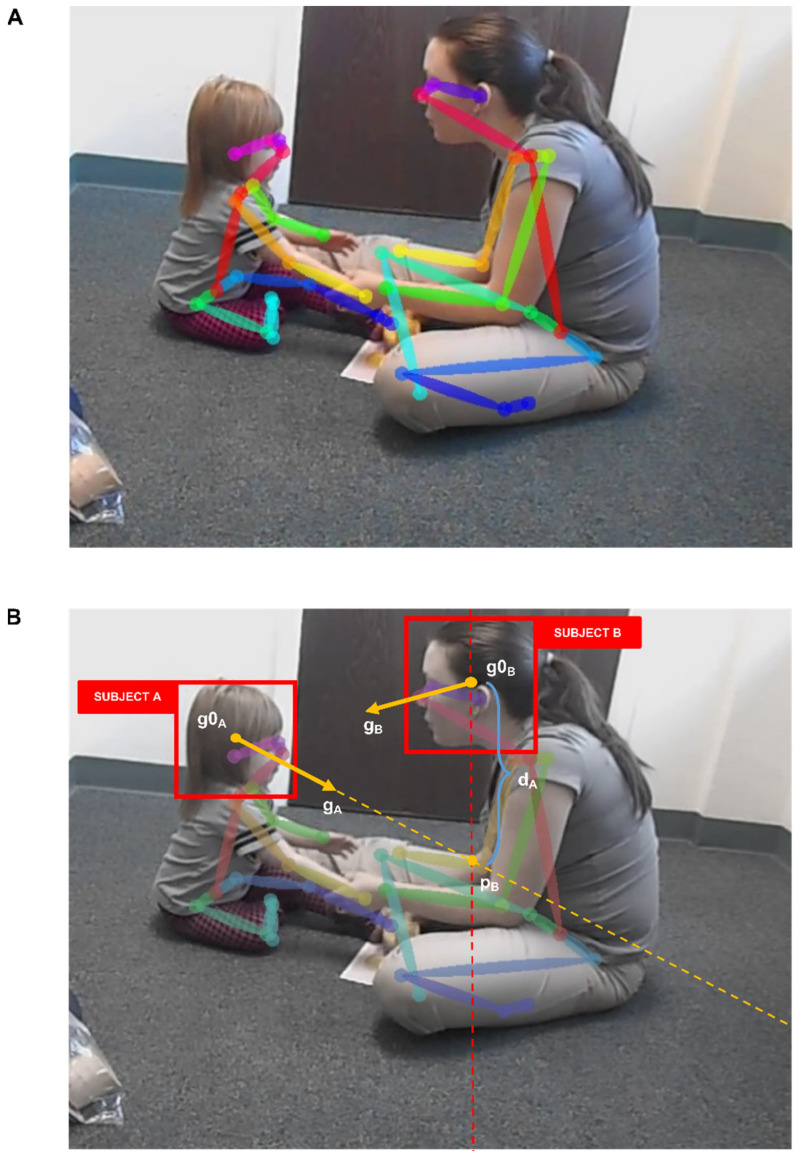
Eye-contact detection model (EYE-C). The images represent the output of the model run on a video example from YouTube (the video is licensed under a CC licence, and was kindly offered by White, R. [Good Behavior Beginnings]. (15 May 2015). *How to Redirect Escape Behavior in 2 year olds* (Video). YouTube. https://www.youtube.com/watch?v=GzGLF8GlPmo, accessed on 28 September 2021); (**A**) OpenPose body keypoints output [47]; (**B**) Gaze360 gaze vectors output [48] and eye-contact detection system; g0_A_: headbox center of subject A; g0_A_/g0_B_: headbox center of subject A/B; g_A_/g_B_: gaze vector of subject A/B; p_A_: intersection point between gaze of subject B and headbox x-axis coordinates of subject A; d_B_: distance (pixels) between p_A_ and g0_A_.

To operationalize this dynamic, we computed the 2D coordinates (x,y) of the intersection p between the line passing through the coordinates of the gaze vector g, and the line passing through the center g0 of the other target’s headbox and perpendicular to the x-axis. Namely, we were able to establish the point p, where the gaze of subject A crossed the position of the head of subject B on the ordinate (Figure 1B). We then calculated the distance d (in pixels) between the intersection point p and the origin g0 to understand the proximity of a subject’s gaze to the target head, as follows:

(1) $$d_{B}=\left|\;p_{B}-g0_{A}\right|$$

The smaller the distance d with respect to the therapist’s head, the more the child’s gaze will be oriented towards the face. To understand whether the child was looking at the adult’s face, we established a maximum distance threshold T_d_ (in pixels). We adopted a threshold rather than the precise center to attempt to contain slight inaccuracies in gaze prediction.

When both distances d_A_ and d_B_ were below threshold T_d_, we would potentially get eye contact. However, this first step is constrained to a bidimensional representation of data. The outputs of OpenPose are two-dimensional coordinates of the landmarks [47]. On the other hand, Gaze360 provides a three-dimensional vector [48]. An issue of considering only the 2D coordinates is to recognize as episodes of eye contact some moments without such coordination, for instance, situations when the subjects’ heads are located at very different depths or more often when they are very close to each other. Neglecting depth may result in many scenarios where gaze directions appear to cross, but only from a 2D perspective. This approach would lead to include several false positives in the analysis and compromise the quality of the data, as well as lose information.

To address this problem, we used a simple but effective approach. The output of Gaze360 is a 3D vector in which depth is expressed through a z value that varies between −1 and 1 [48]. When z assumes a negative value, the subject is looking toward the camera, conversely, it assumes a positive value. Whenever child and therapist look at each other and are on the same depth level in the room, their gaze vectors will have a value of z close to 0. This means the therapist’s gaze will be fully oriented towards the right or left side of the room, and vice versa for the child. On the contrary, when the subjects look at each other from two different room depths, the z value of the gaze vector of the therapist will start to increase or decrease according to its direction. Similarly, the z value of the child’s gaze vector will change, but with an opposite sign. If z of subject A increases, then z of subject B decreases below 0. This is because if vectors are aligned at different depths, they will always have opposite signs. In this way, to recognize eye contact between child and therapist, both gazes need to be close enough to each other’s head and need opposite depth direction.

Moreover, a rarer situation to consider is when both subjects stay at the same depth position, and z is close to 0. In such circumstances, little fluctuations and errors in gaze prediction might vary vector orientation, compromising the analysis and including possible false negatives. To solve this problem, we again established a threshold T_z_ by setting a maximum degree of tolerance for the absolute value of z. When z was close enough to 0, and therefore the gaze directions had nearly no depth, it was unnecessary for the two gaze vectors to have opposite signs. In this way, we were able to control all cases in which subjects were looking at each other closely and at the same depth level of the room. Finally, to prevent the analyses from being affected by false positives, we established a minimum duration threshold (defined as the number of consecutive frames) of eye contact events.

In summary, conditions for discriminating eye contact periods included that (1) the gaze vectors were both oriented toward the headbox of the other within a threshold distance, (2) that the vectors had opposite directions when the absolute value of z exceeded a certain value, and (3) the eye contact events had a minimum duration. This pipeline enables a dyadic eye contact detection system resilient to common variations in terms of video resolution and ecological clinical setting, with enough flexibility to handle interactions with high levels of dynamism.

#### 2.4.2. Model Evaluation

Following the design and method definition part, we evaluated the performance of EYE-C using manual annotations.

Due to the time-consuming hand-coding, we divided the model evaluation into two steps. (1) First, we qualitatively inspected the model’s performance through rendered videos (with gaze vectors, headboxes, and eye contact) to assess in what conditions EYE-C was noticeably failing. In this way, we directly excluded 13 videos recorded with low resolution (384/640 px) and in larger rooms.

(2) Next, we did a quantitative validation by manually coding segments in 5 videos taken from the remaining sample in other conditions. The 5 videos were selected from the sample after matching different resolutions and room settings. We extracted a continuous interactive dyadic sequence of about 10 min was extracted from each considered video, selecting the first sequence with at least 10 episodes of eye contact (at least 1 per minute, to have sufficient comparison data), for a total of more than 70,000 frames (M = 14,173.4, SD = 261.7 frames for video) for the model testing (a total of 4360 positive frames, labelled with eye-contact).

The 10-min videos were subsequently hand-coded frame-by-frame using a software for observational video coding (BORIS, https://github.com/olivierfriard/BORIS, accessed on 25 April 2021). The interactive periods in which there was eye contact between therapist and child were annotated with a binary outcome (eye-contact present/absent) for each frame. As a result, we were able to collect a total of 4360 positive frames and 61 (M = 12.2, SD = 1.7) eye contact events for comparison and model testing. In parallel, the same sequences were further encoded using the eye-contact detector according to the pipeline described in the previous sections.

To assess whether the subset used for validation was representative of the sample, we performed a Kolmogorov-Smirnov test to compare the variance of gaze features between the validation subset and the other 80 videos in the ASD sample (Table S1 in Supplementary Materials). No significant difference emerged for the average duration (dur, *p* = 0.659) and distance (d, *p* = 0.852) of eye contact episodes, but a significant difference was found for frequency (freq, *p* < 0.001). Thus, the validation subsample is representative of the overall sample in terms of duration and distance of eye contact events, but not in terms of frequency. This issue is influenced by considering sub-sequences with a high number of eye contacts (at least 1 per minute) in order to have a higher number of positive annotated frames for validation.

Finally, the output of the model was evaluated using the ground truth annotation labels (eye-contact present/absent) for each frame as reference. The parameters described in Section 2.4.1. (T_d_, T_z_, minimum duration) were empirically selected to maximize matching using the average Matthews Correlation Coefficient (MCC) to evaluate the model’s performance [69].

Overall, the best performance was achieved by using a maximum distance threshold T_d_ corresponding to 80% (d ≤ T_d_) of the target headbox size, a depth threshold T_z_ = 0.3 $\left(\left|z\right|\leq \;0.3\right)$, and a minimum duration of 25 consecutive frame (≃1 s) for eye-contact sequences. The results are described in Table 3, for increasing size of the videos.

The performance of EYE-C was poor for the video sequences with the lowest resolution (384 px), resulting in an MCC = 0.46. In particular, this subset showed a good recall = 0.71 but a very low precision = 0.34, which indicates a high rate of false positives in the results. The setup with 384 px resolution was then excluded by the analysis. The model performed well across all the other conditions with an average MCC = 0.74.

In summary, following the qualitative (1) and quantitative (2) validation phases, we excluded videos recorded in the two larger rooms with resolutions of 384 and 640 px and we excluded videos recorded in the two smaller rooms with a resolution of 384 px. After the procedure, we excluded 23 participants from the analysis, and the sample was accordingly reduced from 85 to 62 subjects. The median sizes of the headboxes extracted from the videos in the sample were 39 × 39 px for the excluded videos and 54 × 54 px for the videos included in the analysis.

Finally, EYE-C was run on the filtered dataset after the evaluation to extract fine-grained features of the eye contact periods between therapists and children with ASD. The metrics applied in the subsequent analyses included: average duration of eye-contact events (M = 1.7, SD = 0.3 s), expressed in seconds (sec) and calculated by dividing the number of frames by the frame rate of the videos (25 fps); average distance d (M = 428.9, SD = 387.5 px), expressed in pixels (px) and calculated by collecting the distance of the child’s gaze from the center of the therapist’s headbox; the total number of eye-contact events (M = 20.4, SD = 19.9); and the frequency of the eye-contact episodes freq (M = 0.4, SD = 0.3), expressed in number per minute.

### 2.5. Data Analysis Plan

For dataset analysis, we considered the eye-contact features as independent variables: average duration (dur), total number (num) and frequency (freq, number per minute) of eye-contact episodes, and the overall average distance d of the child’s gaze from the therapist’s face during the interaction.

As dependent variables, we included the scores of psychological testing from the clinical evaluation. Concerning cognitive functioning, we considered the general developmental quotient (GQ) and 5 relative subscales: gross motor (Motor), hand-eye coordination (Coordination), communication (Language), social (Social), and performance (Perform) abilities. Also, we included the ADOS-2 total raw score (ADOS) and the score of the social abilities subscale (SA) for the socio-communicative dimensions.

We did not consider the single item scores of the ADOS-2, as they are rated on a qualitative scale with little variance (0–2). Indeed, in our sample, 78 (91.8%) of the 85 subjects received the maximum score (=2) in the item related to gaze modulation during the first evaluation. Therefore, we chose to keep only the overall raw score of the ADOS-2. Accordingly, we considered developmental quotient (GQ) for cognitive functioning and raw ADOS-2 scores (ADOS) for social-communication skills within the re-evaluation phase of the intervention sub-sample.

The analysis procedure was further divided into two separate parts: correlation and stratification.

Correlation—As a first step, we explored the correlation between our eye contact features and the clinical variables. We first converted all variables into z-scores to normalize the standard deviation to 1 (using Standard Scaler from the scikit-learn library). Subsequently, we employed multiple linear regressions (MLRs) and checked the assumptions to analyze the relationship between the independent variables jointly against each dependent variable.

Stratification—To investigate spectrum heterogeneity, we then employed unsupervised clustering based on eye-contact features. We first standardized the variables and then employed Uniform Manifold Approximation and Projection (UMAP) for manifold learning and dimensionality reduction. Further, for clustering, we employed Hierarchical Density-Based Spatial Clustering of Applications with Noise (HDBSCAN), which assumes clusters based on density regions and leaves scattered background classified as noise. HDBSCAN is a suitable algorithm for data-driven approaches because it does not need to determine the number of clusters a priori and thus is more efficient in exploratory analysis design than deterministic partitioning algorithms such as K-Means. Finally, we externally validated the clusters by testing for differences in clinical variables based on the resulting sub-groups. We applied a one-way MANOVA using cluster membership as an independent variable. Next, we applied one-way ANCOVAs, with video length and resolution as covariates, and Tukey’s tests for post hoc analysis, pairwise comparisons, and an adjusted *p*-value. Finally, we further analyzed the duration and number of eye-contact episodes over time in a mixed design to see if there were any differences between the sub-groups over the course of the interaction.

## 3. Results

### 3.1. Correlations

Before proceeding with the definition of MLRs, we checked for the assumptions. Multicollinearity occurs when you have two or more independent variables that are highly correlated with each other. We computed the Variable Inflation Factor (VIF) to determine the correlation between eye-contact features by obtaining a score for each variable of how well it is explained by the others.

A VIF score above 5 indicates high multicollinearity. As expected, a strong correlation between frequency and number of eye-contact episodes was found (Table 4). For these reasons, we decided to eliminate frequency (freq) and keep number (num) in the following analysis.

In addition, to have more control over the independent variables, we applied Pearson’s Correlation Coefficient to control the association between the eye-contact features and the length of the videos to avoid a bias due to the duration of the interactions. No significant correlation emerged.

Then we checked the distributions of the variables to check that they followed a normal distribution. We initially used Q-Q plots to test the distribution of the variables. From a visual inspection of the diagnostic plots, the distance (dist) and number (num) of eye contacts, and the communication abilities quotient (Language) did not follow a normal distribution (Figure S1 in Supplementary Materials).

We converted the two variables with a logarithmic transformation. Then we conducted Shapiro–Wilk tests to check for normality of distributions. All variables resulted normally distributed after the conversion (Figure S2 in Supplementary Materials). Next, we standardized all the measurements and computed an MLR for each dependent variable, using our eye-contact features as independent variables (excluding frequency) (Figure S3 in Supplementary Materials). The assumption of homoscedasticity is that the residuals are equal for all values of the predicted dependent variable (i.e., the variances along the line of best fit remain similar as you move along the line). We checked for homoscedasticity by controlling the plots of studentized residuals versus unstandardized predicted values and by performing the Breusch–Pagan test for heteroscedasticity (Figure S4 in Supplementary Materials). For each of the MLRs, homoscedasticity of the residuals was confirmed by visual inspection and non-significant test results. Finally, we checked the distribution of residuals by again using the Shapiro–Wilk test and found the normality of the error distributions for all MLRs (Figure S5 in Supplementary Materials). The results of all MLRs for each of the dependent variables are summarized in Table S2 in Supplementary Materials.

A significant regression equation with a non-robust negative correlation was found for the ADOS-2 total score (F(3,58) = 2.718, *p* < 0.05), with an R^2^ = 0.123, and the related Social Abilities subscale (F(3,58) = 2.866, *p* < 0.05), with an R^2^ = 0.129. No significant regression was found for the general developmental quotient, but a significant regression equation with a non-robust positive correlation was found for the related subscales of communication (F(3,58) = 2.795, *p* < 0.05), with an R^2^ = 0.126, and hand-eye coordination (F(3,58) = 2.783, *p* < 0.05), with an R^2^ = 0.126, abilities. All remaining MLRs for the subscales of the GQ were non-significant.

The average duration (dur) and distance (d) of eye contact episodes were not significant predictors for all regression models. Otherwise, the total number (num) of eye contacts during child-therapist interactions was a significant predictor of ADOS (*p* < 0.01), SA (*p* < 0.01), Language (*p* < 0.05), and Coordination (*p* < 0.05) scores.

### 3.2. Stratification

The second part of the analysis design further explored the findings by investigating the effectiveness of gaze patterns (num, dur, freq, d) for spectrum stratification and identifying the possible occurrence of sub-groups within the sample. Given recent findings regarding the importance [30], we also considered age as a factor for clustering. We initially normalized the variables by converting them into z-scores.

**Figure 2:**
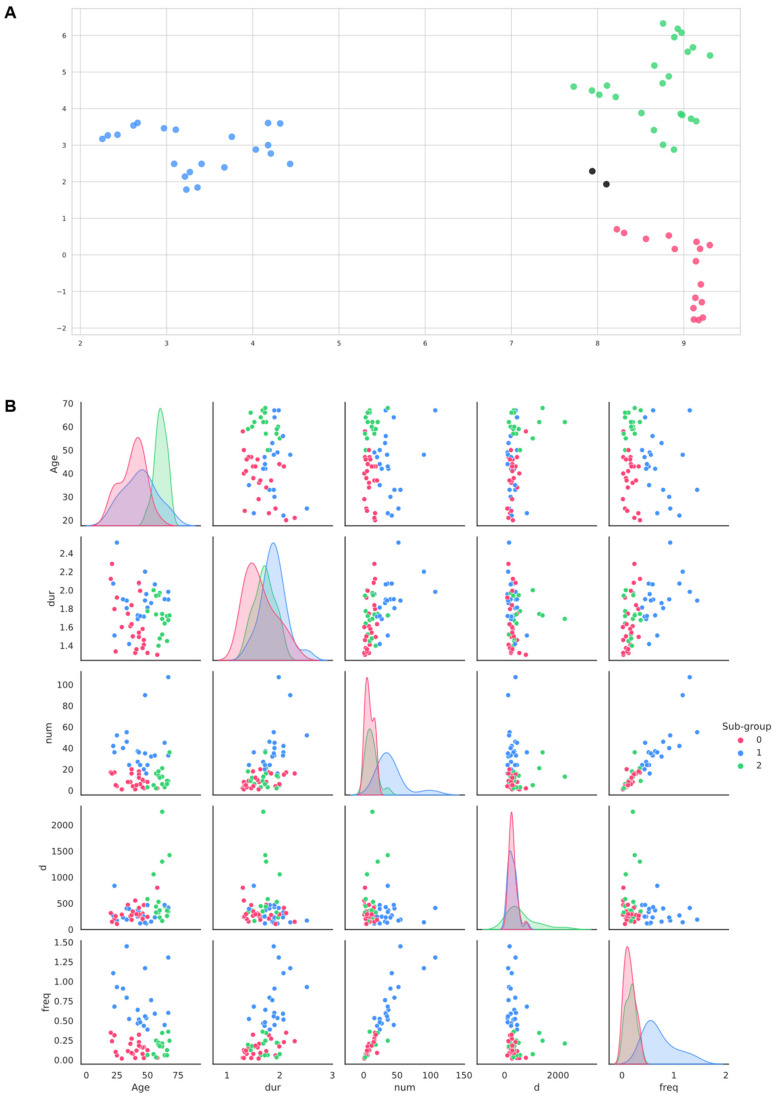
(**A**) HDBSCAN clusters on 2-components UMAP output, the 2 black marks represent the single data points classified as noise; (**B**) Eye-contact metrics and age pairplot; freq: frequency of eye-contact episodes; num: total number of eye-contact episodes; dur: average duration of eye-contact episodes; d: average distance of children’s gaze vectors from therapists’ headboxes during interaction; age: children’s age at first assessment.

We employed the uniform manifold approximation and projection (UMAP) for non-linear dimensionality reduction and improved data visualization [70,71]. A 2-component UMAP was applied on the 4 scaled eye-contact features and age jointly to reduce data structure using 5 nearest neighbors and a minimum distance of 0 as hyperparameters of the algorithm. The output is a 2D projection of the data structure into low-dimensional space, based on a transformation (embedding) of the selected features (Figure 2A). The resulting data embedding was further processed for unsupervised clustering.

We used the HDBSCAN algorithm, setting a minimum cluster size of 10, equal to twice the number of features used (2 × 5 dim) [72]. In the dataset, three different clusters were identified (sub-groups 0/1/2), and two single data points were classified as noise (sub-group-1) (Figure 2A,B).

We computed the silhouette coefficient (SC) to assess the consistency and homogeneity of the resulting clusters and achieved a score of SC = 0.56. The characteristics and population size of the clusters are summarized in Table 5.

The sub-groups were first compared according to control variables and showed no significant differences in gender, video resolution, room setting, and duration of therapist-child interaction (Table 5).

**Figure 3:**
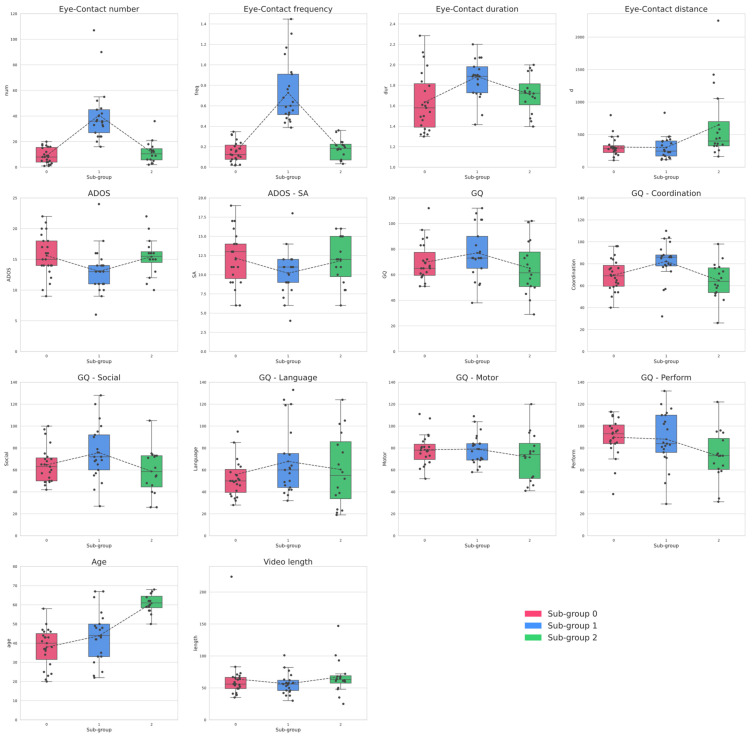
Boxplots of each dependent and independent variable for resulting sub-groups; num: eye-contact periods total number; freq: eye-contact periods frequency (N/min); dur: eye-contact periods average duration (s); d: average distance of children’s gaze vectors from therapists’ headboxes during interaction (px); age: children’s age at first assessment (months); length: video total duration (min).

Finally, clustering was evaluated by comparing the differences between the groups, considering both clinical and eye-contact features (Figure 3). A one-way MANOVA was applied with group membership as the independent variable and clinical metrics as the dependent variables. A statistically significant difference emerged between the sub-groups on the combined dependent variables, (F(13,46) = 6.393, *p* < 0.0001).

As follow-up analyses, univariate one-way ANCOVAs and Tukey’s Tests were performed for post hoc pairwise comparisons of each dependent variable.

Concerning our eye-contact features, we found a significant difference in total number (F(2,57) = 31.82, *p* < 0.0001), frequency (F(2,57) = 55.577, *p* < 0.0001), average duration (F(2,57) = 5.815, *p* < 0.01), and the overall average distance (F(2,57) = 6.618, *p* < 0.01) between the sub-groups. A significant difference between the sub-groups was also found in terms of the children’s age (F(2,57) = 23.597, *p* < 0.0001).

Comparing the sub-groups based on clinical variables revealed significant differences concerning the ADOS (F(2,57) = 3.549, *p* < 0.05) total score and the cognitive development subscales of social (F(2,57) = 3.207, *p* < 0.05), hand-eye coordination (F(2,57) = 5.803, *p* < 0.01), and performance (F(2,57) = 3.238, *p* < 0.05) abilities.

We further tested the significant results achieved through pairwise comparisons with an adjusted *p*-value (Table S3 in Supplementary Materials). Tukey post hoc tests showed that sub-group 1 showed a significantly higher number and frequency of eye contact than both sub-groups 0 (num *p* < 0.001, freq *p* < 0.001) and 2 (num *p* < 0.001, freq *p* < 0.001). Conversely, sub-groups 0 and 2 did not differ either in number (*p* = 0.82) or frequency (*p* = 0.9). In terms of duration, sub-group 1 showed significantly longer episodes of eye contact than sub-group 0 (*p* < 0.01), but no difference emerged either between sub-groups 1 and 2 (*p* = 0.096) or 0 and 2 (*p* = 0.595). In addition, sub-group 2 showed a significantly higher overall distance d than both sub-groups 1 (*p* < 0.01) and 0 (*p* < 0.01); the latter showed no difference in distance (*p* = 0.9).

Regarding the clinical variables, a difference emerged between the ADOS-2 total scores of sub-groups 0 and 1 (*p* < 0.05), the latter having significantly lower scores. In contrast, there were no significant differences between the other sub-groups (2 vs. 0 *p* =.9, 2 vs. 1 *p* = 0.1).

In terms of hand-eye coordination abilities, sub-group 1 showed a significantly higher mean quotient compared to both sub-groups 0 (*p* < 0.05) and 2 (*p* < 0.01), whereas sub-groups 1 and 2 did not differ (*p* = 0.063). Sub-group 1 showed a significantly higher score in the social abilities subscale than sub-group 2 (*p* < 0.05), but not sub-group 0 (*p* = 0.195); there was no difference between sub-groups 0 and 2 (*p* = 0.653). Concerning the performance subscale, pairwise comparisons showed no significant differences among sub-groups.

When comparing age, sub-group 2 was significantly older than sub-groups 1 (*p* < 0.001) and 0 (*p* < 0.001), which also did not differ in age (*p* = 0.152).

We also compared the three sub-groups on the basis of the resolution of the videos, the duration of the video, the room where the interaction occurred, and the gender of the subjects as controls and found no significant differences.

In addition, to better explore the characteristics of the subgroups, we tested for the presence of outliers in the clinical variables (GQ, GQ subscales, ADOS, SA subscale) by converting data into z-scores and detecting specific cases deviating over 3 standard deviations from the mean. In sub-group 1, an outlier emerged showing eye contact metrics (num = 37, freq = 0.6, dur = 1.7 s, d = 365 px) and age (=42 months) consistent with the sub-group average, but a higher (z = 3.02) ADOS score (=24). In subgroup 0, an outlier emerged showing metrics of eye contact (num = 15, freq = 0.2, dur = 2.1 s, d = 413 px) and age (=43 months) in the subgroup average, but a higher score (z = 3.71) in the subscale of language abilities (=152).

As the last step of clustering exploration, we divided interactions into 4 equivalent time-points to check whether there was any difference in gaze patterns over time between the sub-groups. We measured both the number and duration of eye contact periods at 4 consecutive time points. Mixed two-way ANOVAs were performed with number and duration over time as the within factors and sub-group membership as the between factor (Figure S6 in Supplementary Materials).

As expected from the previous analyses, there was an overall significant difference between the sub-groups in both number (*p* < 0.0001) and duration (*p* < 0.0001). There was no significant difference within the number (*p* = 0.195) and duration (*p* = 0.246) of eye-contact episodes over periods of the interaction. Finally, there was no significant interaction between the sub-groups and the duration (*p* = 0.41) or number (*p* = 0.725) over time.

## 4. Discussion

The study aimed to develop and test an efficient computational phenotyping method to study the interactive behavior of young children with ASD for ecological exploration of gaze patterns during therapy. Identifying a marker sensitive to individual differences is an important goal in the perspective of personalized treatment. Despite promising results in the literature, the major bottleneck is the development of generalizable and flexible methods into real-world scenarios. For this reason, we implemented a method that is resilient to variability in data structure with added applicative value. Our approach combined unsupervised machine learning analysis with a data collection based on fine-grained features acquired by behavior imaging solutions.

Eye contact was studied both for its central role in the diagnostic framework as well as its value as an indicator of the severity of social-communicative symptoms in the autism spectrum [21,22,23,28,30,43,46]. Indeed, it has recently been suggested that attentional patterns should be further investigated in the context of outcome prediction [46] and stratification of the condition [30,43].

The first part of our study addressed the development of EYE-C, the eye-contact detection model. Our main goal was to offer a more practical solution trying to overcome the translational limitations of previous implementations of CV-based systems in the clinical setting [17,18]. Towards this end, we implemented a state-of-the-art model-based design for behavior analysis in wild videos [47,48]. Our system performed well during validation in clinical scenarios with an average MCC = 0.74 across different interaction videos, with different resolution and setting. We were able to identify episodes of eye contact with good precision and accuracy in highly dynamic interactions between child and therapist. To the best of our knowledge, ours is the first solution for eye contact detection in non-structured clinical settings.

We implemented a method to deliver more reliable and quantifiable measurements of a behavioral feature that is very important in clinical ASD. Previously developed solutions were based on a heavy structuring of interaction, which often limited the value of subsequent analysis and application aspects [11,32,35,36,37,38,39,40,41]. Considering the importance of integrating eye contact into intervention programs [21], our method offers a solution with the potential to support the real clinical context of ASD. Our system can better decompose the dynamics of gaze and eye contact than commonly used testing techniques, i.e., ADOS-2 scores, which are not suitable to accurately quantify behavior.

In the second part of the study, we employed EYE-C to explore the dynamics of dyadic gaze coordination in child-therapist interactions. The need for more refined measurements of behavior has been highlighted in the literature to address major challenges such as stratification and outcome prediction [9,73]. Traditional psychological testing, which is generally used in this area of research, is rather validated to diagnose and detect differences from typical development. Therefore, it is not well suited to recognize the subtle variability within the spectrum [7,9,74]. In our study, more than 90% of the sample had the same maximum score in the item of ADOS-2 concerning eye contact abnormalities. Behavior Imaging offers an excellent opportunity in this perspective by allowing quantitative and refined measurements to study behavior in a more systematic way [11]. Consistently, in the present work, we have employed eye-contact features collected in this manner combined with data-driven analysis of unsupervised machine learning.

The first step of the analysis covered a preliminary exploration of the gaze features extracted. We examined the correlation between our metrics and the clinical variables collected during the assessment of preschool children with ASD. The results of the regression models confirmed the presence of associations, although not robust, between the eye-contact features and the rates of symptom severity, and some subscales of cognitive functioning. As hypothesized, a negative correlation emerged between ADOS scores together with the relative subscale of social impairments (SA) and the number of eye contact episodes. Children with a higher degree of interactive deficits and higher severity of social symptoms displayed less eye-contact coordination with the therapist. These data are consistent with findings in the literature regarding the association between the degree of attention to the adult face and the severity of autistic symptoms in preschool children [46]. No correlation was found concerning general cognitive functioning, suggesting a stronger association between attentional patterns and socio-interactive aspects, rather than cognitive ones. However, taking into account the individual subscales, positive correlations emerged within the domains of hand-eye coordination and communication abilities. These findings are also coherent if we consider the importance of motor coordination aspects in the integration of attentive schemas and the critical role of eye contact in the later development of socio-communicative skills [21,29,75,76]. Contrary to expectation, there was no significant association with the social abilities subscale. This might be explained in part by considering that the social subscale of the GMDS-ER includes both items related to interactive skills and items related to the child’s level of autonomy, which is less related to social abilities; yet this will need to be further investigated.

In the second stage of data analysis, we stepped forward to address the challenge of autism heterogeneity. We employed unsupervised clustering based on eye-contact features to check whether sub-groups would emerge within the spectrum. Unsupervised approaches applied to computational phenotyping outputs can also facilitate the development of fine-grained instruments and the identification of novel specifiers that may help detect reliable subtypes. Along with attentional metrics, we also considered age as a factor, given the findings regarding its importance in stratification [30]. Three different homogeneous clusters were found, which differ in gaze coordination and age. Sub-group 1 (high-coordination) is characterized by including toddlers who showed improved gaze coordination, including a higher number, frequency, and duration of eye contact episodes. In contrast, the other two sub-groups (low-coordination, 0 and 2) were characterized by lower and similar eye contact features. Considering age, the low-coordination cluster 2 (old-low-coordination) was distinguished by including children with significantly higher age than the low-coordination cluster 0 and the high-coordination cluster 1.

To summarize, two low- and high-coordination sub-groups (0 and 1) of age-matched toddlers were found, which differed significantly in terms of number, frequency, and duration of eye-contact episodes with the therapist during the interaction. In addition, a smaller third old-low-coordination sub-group (2) was identified, which was characterized by a quality of gaze coordination comparable to the other low-coordination sub-group, but at a higher age. Interestingly, the children in the old-low-coordination cluster also differed from the others in displaying a higher overall distance d, which measures the distance of the child’s gaze from the face of the therapist. This may be explained by the fact that in typical development, older children generally tend to be less focused on dyadic interaction and explore the environment more by gazing at objects and paying less attention to adults in general.

Afterward, the clustering was validated by taking into account the clinical variables collected during children’s diagnostic assessment, which included both symptom severity (ADOS-2, SA), and level of functioning scores (GQ, Coordination, Social, Motor, Language, Perform). When comparing the clinical characteristics of the sub-groups, the high-low distinction remained consistent, with high-coordination children showing higher levels of general cognitive functioning, social abilities, and hand-eye coordination along with lower scores on the symptom severity and social impairment. In comparison, the two low-coordination clusters showed comparable clinical features, including a higher degree of symptom severity and social impairments and lower cognitive functioning, social abilities, and coordination.

In a statistical analysis of the distributions, the two age-matched sub-groups of low- and high-coordination were significantly distinguished for symptom severity and hand-eye coordination abilities. The older low-coordination cluster did not differ in any clinical variable from the other low-coordination sub-group but showed differences in hand-eye coordination and social abilities compared to the high-coordination sub-group.

Altogether, some interesting data emerged from stratification. Our findings seem to support the hypothesis that the autism spectrum could be stratified into two major levels of functioning, consistent with what was found in previous studies [5,43,77]. Two core age-matched sub-groups emerged, one cluster consisting of autistic children with a milder symptom phenotype, better hand-eye coordination skills, and showing a higher number and duration of eye-contact episodes with the therapist, while the other cluster included autistic children with lower eye-contact features, lower hand-eye coordination abilities and a higher degree of symptom severity.

When observing data distributions, high and low functioning profiles also remained stable in the three sub-groups across the social impairments subscale of the ADOS-2. Nonetheless, no significant differences emerged as we expected, but further investigation is necessary. In addition, differences in hand-eye coordination abilities were also found significant between the high-coordination and the old-low-coordination sub-groups. These persistent differences across clusters corroborate the results of prior analyses and are consistent with previous studies that supported a strong link between fine-motor coordination and social competencies [75,76,78,79,80]. From an operational outlook, given the importance of eye contact in early intervention and its potential for predicting outcomes [30,43,46], we could hypothesize that identifying clusters of children with ASD and worse gaze coordination could assist clinicians in focusing treatment activities on specific aspects of interaction. This suggests that children who show increased eye contact impairments also have higher symptom severity and lower hand-eye coordination abilities. Furthermore, from a longitudinal perspective, it would be interesting to monitor eye-contact features across intervention sessions and to investigate the characteristics of the clusters in which we found concerning developmental trajectories and intervention outcomes.

Overall, this work highlights once again the major potential of behavior imaging for the analysis of behavior in clinical practice [11,12]. To provide concrete support, it is necessary to develop robust, translational approaches that are flexible to the dynamics of interaction and that take into account the variability of settings [18]. In our study, structuring an effective system to measure gaze patterns in a refined and ecological way yielded interesting results in the field of stratification within the autism spectrum. A flexible analytical system was developed by employing advanced AI-based models with high potential for translational applications to real-world clinical scenarios. Computational solutions could help integrate aspects of heterogeneity, paving the way for personalized treatment based on individual differences [6,14,15,16]. This may assist clinicians in optimally delivering intervention based on (1) quantitative data, (2) fine-grained analysis impossible to be carried out by humans involved in the social interplay [81]. From a clinical standpoint, this approach may support the implementation of better-tailored interventions aimed at maximizing efficacy in terms of developmental outcomes, symptom severity reduction, and adaptive functioning [8,14,16,18].

Our research represents an initial step towards this perspective by trying to develop a methodology with a more applicative value. By exploring the validity of systematic gaze features in categorizing spectrum heterogeneity, we have highlighted the role of computational applications in the clinical context. In terms of intervention, they could help therapists identify more precise measures to quantify and categorize atypical behaviors and deliver timely personalized treatment.

### Limitations

This study also carries some relevant methodological limitations. Firstly, the limited data size may constrain the generalizability of the results in this study regarding the UMAP-HDBSCAN clustering. In particular, the reported number of clusters tends to grow with an increased sample size [5,21]. In addition, the analysis of the outliers showed the presence of two subjects with borderline clinical profiles as compared to their sub-groups, which need further investigation. It would be thus interesting to verify on a larger dataset if novel additional data may introduce additional subgroups based on eye-contact patterns. Further, the stratification could be analyzed in the context of personalized early intervention, in particular to investigate the role of these variables in outcome prediction. Additionally, including a control sample with typical development would be beneficial. In particular, clarifying whether the correlations that emerged are specifically related to the autistic phenotype or, more generally, to the degree of social impairments would strengthen the analyses and deepen the functional nature of the clustered subgroups. Finally, from a more technical point of view, the validation process of the model was limited by the availability of annotations for five hand-coded videos. Despite a large number of frames was extracted in this comparison (>70,000 frames and >4300 eye-contact examples), and that all the relevant setups were covered, the number of annotated frames is potentially limited compared to the whole sample size. Tests comparing the variance of the gaze features in the validation subset and the general sample showed that although the subset appears to be representative for duration and distance of eye contact episodes, there is a difference in frequency. This difference can be explained by choosing sequences with a higher number of eye contacts to have more balanced data and a higher number of positive events for comparison. Accordingly, it is appropriate to take those aspects into account when interpreting the results.

## 5. Conclusions

The main contribution of this study is the availability of a computational phenotyping system that has potential application in clinical activity. Refined analysis of attentional dynamics provided interesting results in the field of spectrum stratification, yet the findings need to be further investigated on a larger longitudinal dataset. The future validation of similar translational methods may help bridge the gap between research and healthcare environments and considerably enhance clinical practice by offering solutions to support therapists and families in symptom monitoring during the treatment of children with ASD.

## Figures and Tables

**Table 1:** Population characteristics.

	ASD Sample
	*n* = 85
Age (months), mean (SD)	46.32 (13.8)
GQ, mean (SD)	71.54 (17.4)
ADOS, mean (SD)	14.82 (1.4)
Gender, N (%)	
Male	74 (87.1)
Female	11 (12.9)

Note: ADOS: Autism Diagnostic Observation Schedule, 2nd edition, raw score; GQ: Global Developmental Quotient (GMDS-ER); ASD: Autism Spectrum Disorders.

**Table 2:** Video resolution for the ASD sample.

Room Size.	Video Resolution (px)				
	384 × 288	640 × 480	720 × 576	1280 × 720	1920 × 1080
Small, N (%)	10 (11.8)	5 (5.9)	43 (50.6)	1 (1.2)	0
Large, N (%)	3 (3.5)	10 (11.8)	11 (12.9)	0	2 (2.4)

**Table 3:** Model evaluation results.

Video	Frames (N)	Res (px)	Time (min/s)	Room	Acc	Pre	Rec	MCC
1	13,786	640	12′31″	Small	0.96	0.65	0.80	0.70
2	13,955	1280	6′20″	Small	0.95	0.53	0.76	0.61
3	14,317	720	3′29″	Large	0.96	0.79	0.65	0.69
4	14,497	720	9′11″	Small	0.99	0.94	0.94	0.93
5	14,312	384	20′42″	Small	0.93	0.34	0.71	0.46

Note: Time: subsection beginning timing in the original video; Res: Resolution; Acc: Accuracy; Pre: Precision; Rec: Recall; MCC: Matthews Correlation Coefficient.

**Table 4:** Variable Inflation Factor (VIF) results.

Variable	VIF
Freq	5.6
Num	5.42
D	1.07
Dur	1.34

Note: freq: eye-contact periods frequency; num: eye-contact periods total number; d: average child gaze distance d; dur: average eye-contact periods duration.

**Table 5:** Sub-groups characteristics.

	Sub-Group 0	Sub-Group 1	Sub-Group 2	F/χ2	*p*
Clinical sample	*n* = 23	*n* = 21	*n* = 16		
Gender, N (%)				3.572	0.734
Male	22 (95.7)	17 (80.9)	12 (75)		
Female	1 (4.3)	4 (19.1)	4 (25)		
Video resolution (px)	-	-	-	11.069	0.748
Interaction duration (min), mean (SD)	64 (37.1)	56.3 (16.3)	67.5 (28.1)	0.750	0.477
Room setting (small/large)	-	-	-	0.291	0.865
Age (months), mean (SD)	37.9 (10.2)	43.9 (10.1)	60.9 (10.1)		
Eye-contact num, mean (SD)	9 (6.2)	40.7 (6.2)	11.6 (6.1)		
Eye-contact freq (N/min), mean (SD)	0.2 (0.1)	0.7 (0.1)	0.2 (0.1)		
Eye-contact dur (sec), mean (SD)	1.6 (0.3)	1.9 (0.3)	1.7 (0.3)		
Eye-contact d (px), mean (SD)	308.9 (152.1)	301 (152.1)	649.4 (152.1)		
GQ, mean (SD)	69.7 (15.4)	77.1 (15.4)	65 (15.4)		
Coordination, mean (SD)	69.6 (14.5)	82.2 (14.5)	64.8 (14.5)		
Language, mean (SD)	55.7 (26.6)	67.8 (26.6)	60.4 (26.6)		
Motor, mean (SD)	78.4 (13.8)	79.1 (13.8)	71.5 (13.8)		
Social, mean (SD)	64.6 (16.6)	75.8 (16.6)	58.7 (16.6)		
Perform, mean (SD)	89.9 (18)	88.1(18)	72.6 (18)		
ADOS, mean (SD)	15.6 (3.5)	13.1 (3.5)	15.4 (3.5)		
SA, mean (SD)	12.2 (3.5)	10.2 (3.5)	11.8 (3.5)		

Note: freq: eye-contact periods frequency; num: eye-contact periods total number; d: average child gaze distance d; dur; average eye-contact periods duration. GQ: Global Developmental Quotient; SA: Social Abilities subscale.

## Data Availability

The parents of all the participants gave their consent to the publication of the results of the study, anonymously, in aggregated form. Due to ethical and privacy issues, sensitive data cannot be shared.

## Supplementary Materials

The following are available online at https://www.mdpi.com/article/10.3390/brainsci11121555/s1, Supplementary Figure: S1, Q-Q Plots for normality of distributions diagnostics; Figure: S2, Distributions of variables after logarithmic transformation; Figure: S3, Linear regressions of candidate predictors with the EYE-C feature as dependent variable; Figure: S4, Homoscedasticity of residuals; Figure: S5, Residuals distributions; Figure: S6, Eye-contact periods total number (num) over time; Table S1, Kolmogorov-Smirnov test results; Table S2, Multiple Linear Regressions summaries for all dependent variables; Table S3, Sub-groups pairwise comparisons.

## Abbreviations

EYE-C	Eye-Contact robust detection system
ASD	Autism Spectrum Disorders
AI	Artificial Intelligence
GQ	General Developmental Quotient
SA	Social Abilities subscale
CV	Computer Vision
CNN	Convolutional Neural Network
PAFs	Part Affinity Fields
LSTM	Long Short-Term Memory
MCC	Matthews Correlation Coefficient
MLR	Multiple linear regression
UMAP	Uniform Manifold Approximation and Projection
HDBSCAN	Hierarchical Density-Based Spatial Clustering of Applications with Noise
VIF	Variable Inflation Factor
SC	Silhouette coefficient
