# Supplementary material for: EYE-C: Eye-Contact Robust Detection and Analysis during Unconstrained Child-Therapist Interactions in the Clinical Setting of Autism Spectrum Disorders
Source: Brain Sci. 2021 Nov 24;11(12):1555. doi: 10.3390/brainsci11121555 (PMC8699076; doi:10.3390/brainsci11121555)
Supplement: Supplementary file 1 [file brainsci-11-01555-s001.zip › brainsci-1415880-supplementary.pdf]

Supplementary Materials

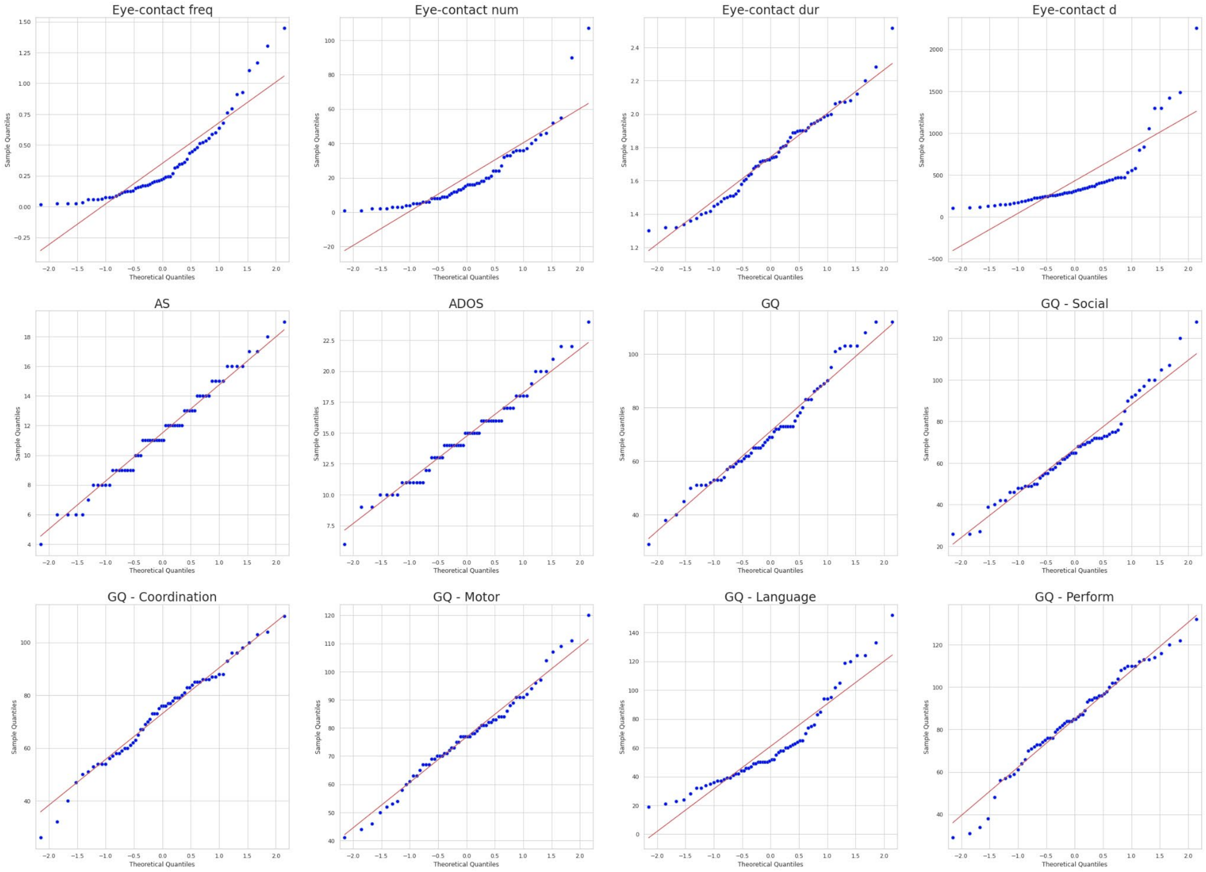

Figure S1. Q-Q Plots for normality of distributions diagnostics.

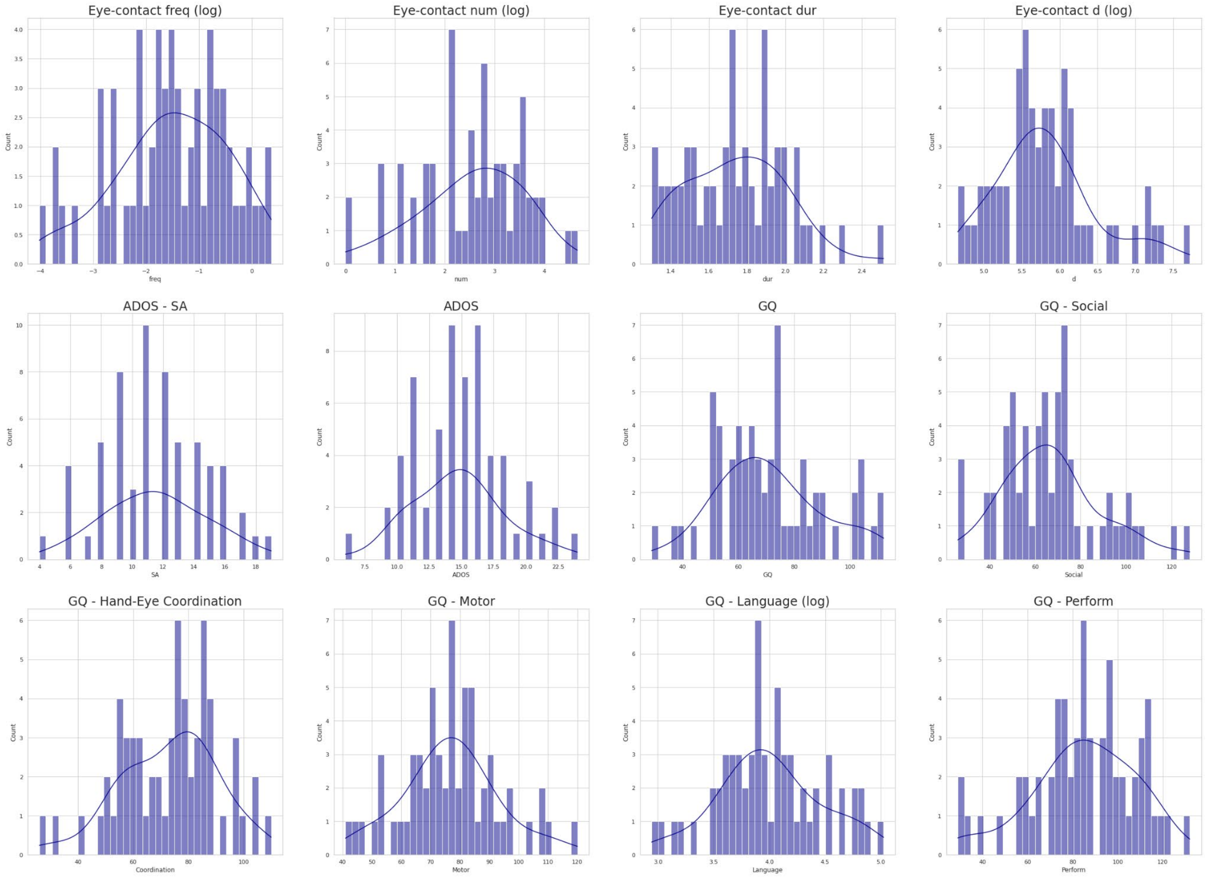

Figure S2. Distributions of variables after logarithmic transformation.

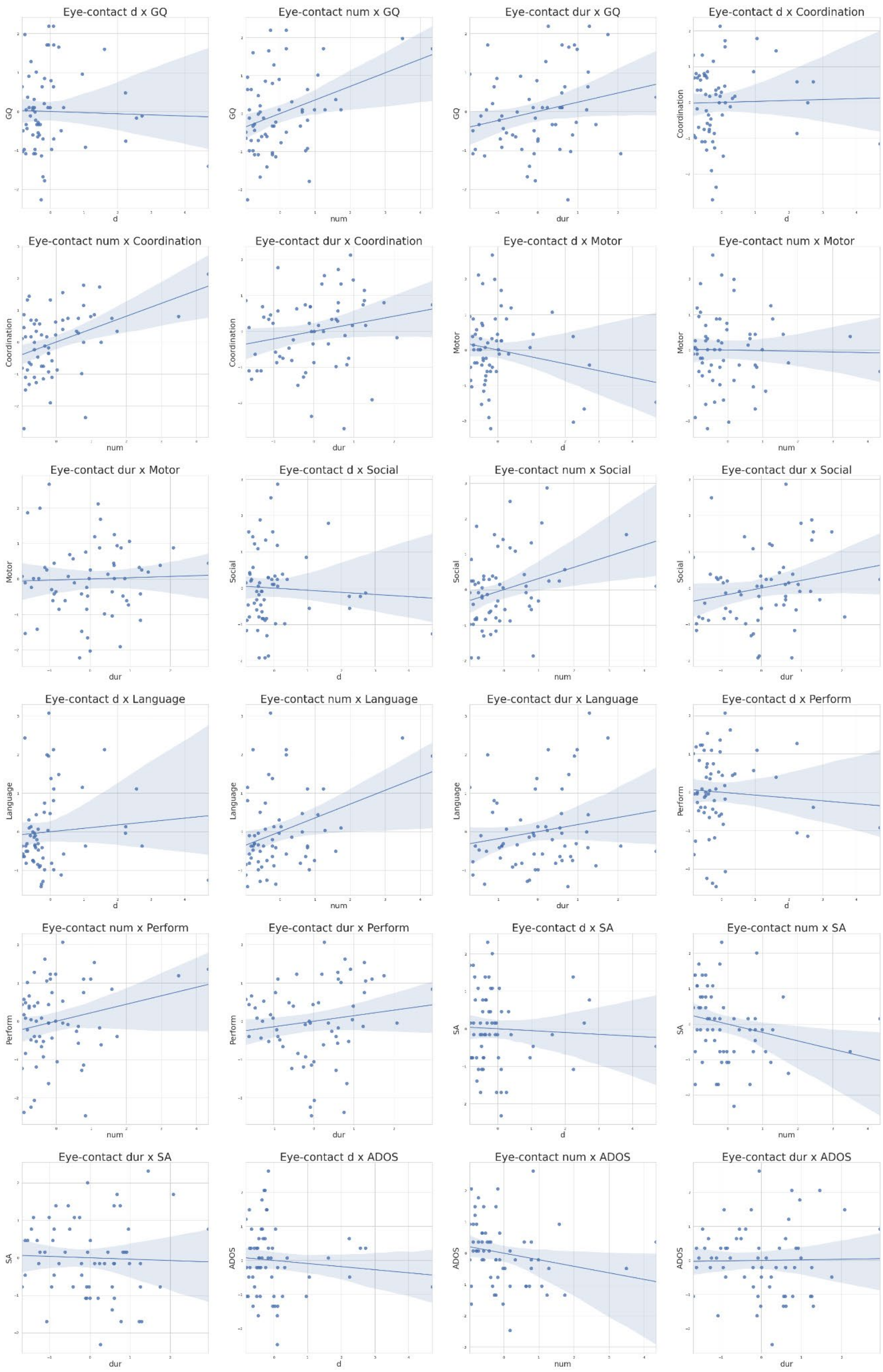

Figure S3. Linear regressions of candidate predictors with the EYE-C feature as dependent variable.

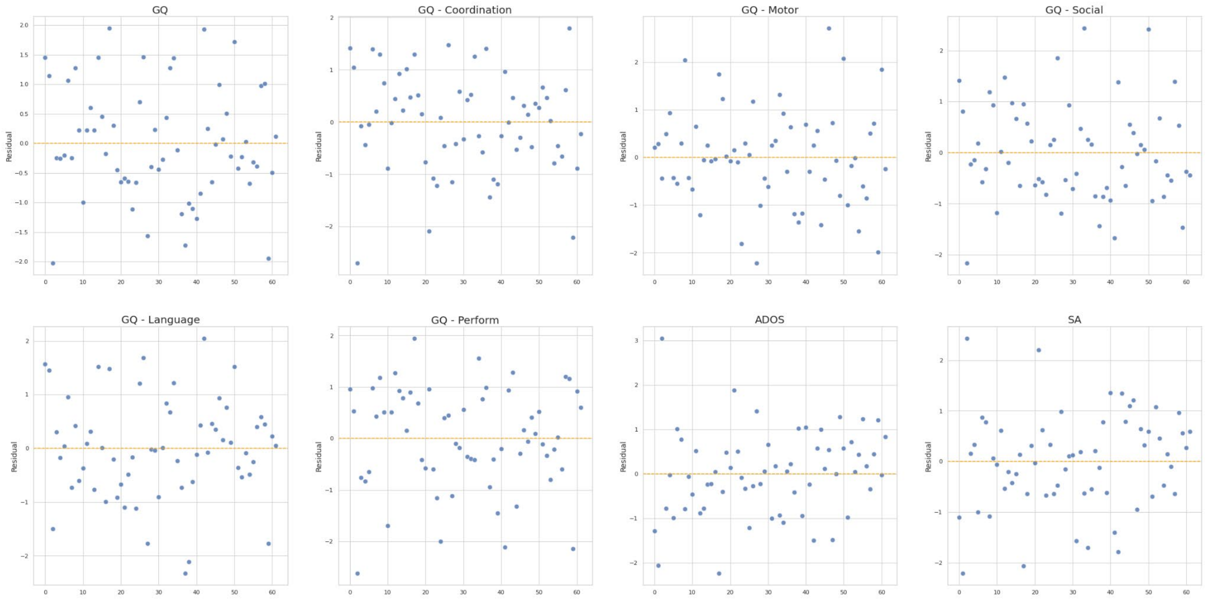

Figure S4. Homoschedasticity of residuals.

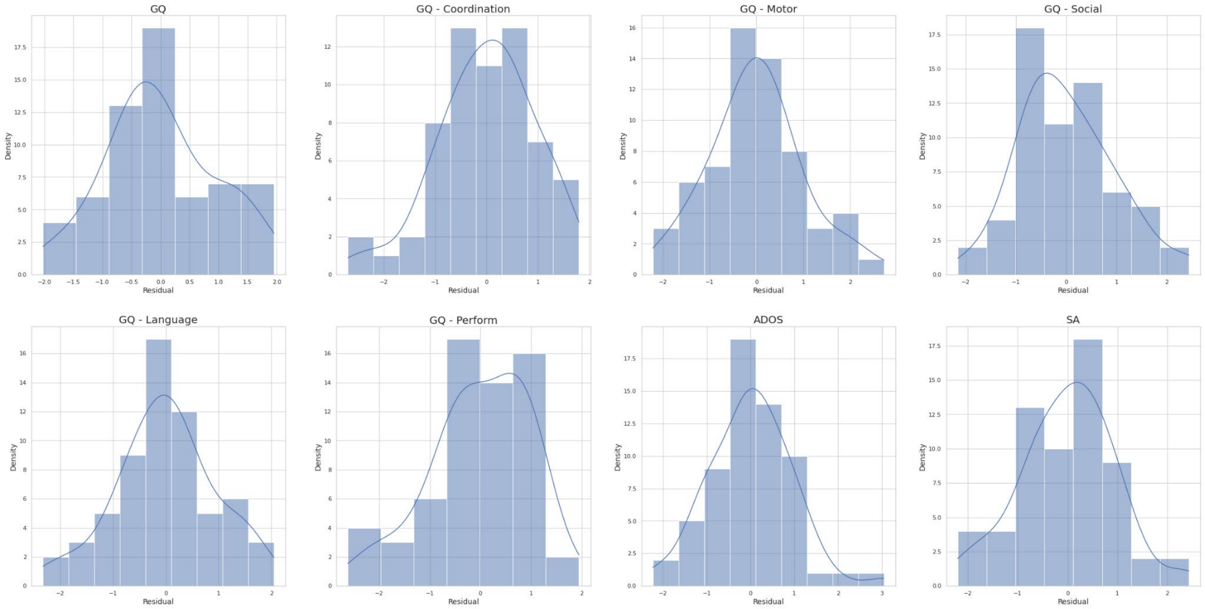

Figure S5. Residuals distributions.

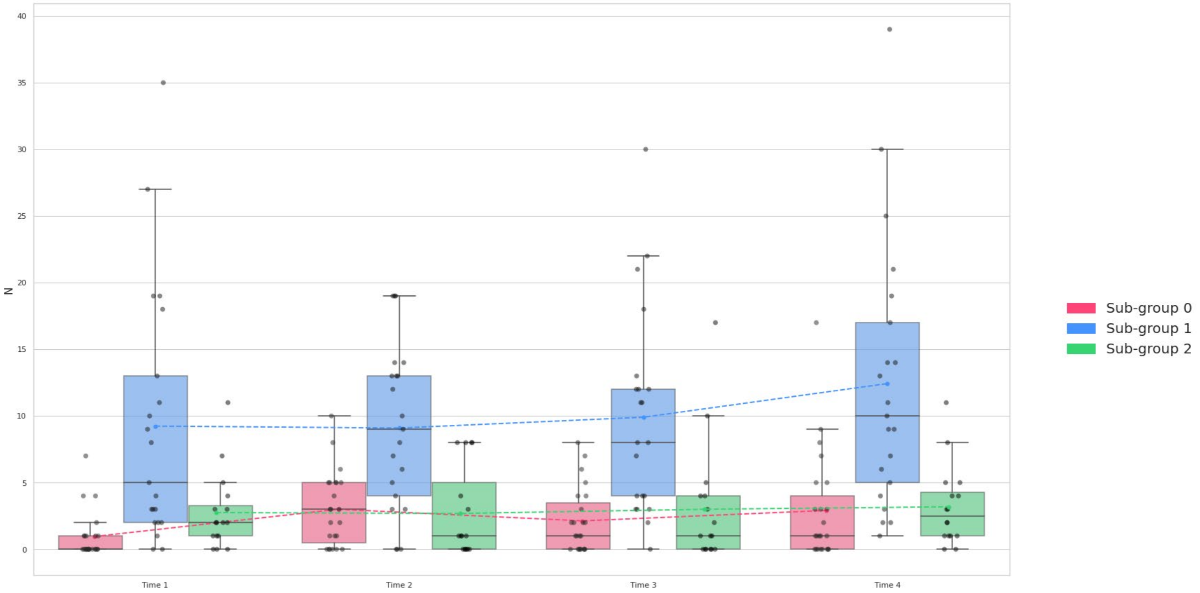

Figure S6. Eye-contact periods total number (num) over time.

Table S1. Kolmogorov-Smirnov test results.

**Variable**	**Validation sub-sample**	**General sample**	**Statistic**	***p***
freq (N/minute), mean (SD)	2.1 (0.8)	0.3 (0.3)	0.963	***
dur (seconds), mean (SD)	1.87 (0.3)	1.72 (0.3)	0.309	0.659
d (px), mean (SD)	340.1 (169.3)	423.9 (387.5)	0.254	0.852

Note: Validation sub-sample: subset of 5 video sections used for model validation; General sample: sample including other 80 participant videos; freq: eye-contact periods frequency; d: average child gaze distance; dur; average eye-contact periods duration.

*p<0.05; **p<0.01; ***p<0.001

Table S2. Multiple Linear Regressions summaries for all dependent variables.

**Variable**		**R^2**	**Adj. R^2**	**F**	**F - prob.**	**coef.**	**t**	**t - prob.**
**ADOS**		0.123	0.078	2.718	*			
	const					0.000	0.000	1
	num					-0.391	-2.758	**
	d					-0.108	-0.865	0.391
	dur					0.191	1.336	0.187
**SA**		0.129	0.084	2.866	*			
	const					0.000	0.000	1
	nu					-0.4	-2.83	**
	m							
	d					-0.106	-0.849	0.399
	dur					0.141	0.991	0.326
**GQ**		0.108	0.062	2.349	0.082			
	const					0.000	0.000	1
	num					0.233	1.631	0.108
	d					0.12	0.952	0.345
	dur					0.143	0.994	0.324
**Coordination**		0.126	0.081	2.783	*			
	const					0.000	0.000	1
	num					0.315	2.22	*
	d					0.099	0.79	0.432
	dur					0.074	0.515	0.609
**Motor**		0.011	-0.04	0.218	0.884			
	const					0.000	0.000	1
	num					-0.03	-0.198	0.844
	d					-0.099	-0.746	0.459
	dur					0.031	0.204	0.839
**Language**		0.126	0.081	2.795	*			
	const					0.000	0.000	1
	num					0.309	2,184	*
	d					0.187	1.492	0.141
	dur					0.042	0.297	0.767
**Social**		0.109	0.063	2.376	0.079			
	const					0.000	0.000	1
	num					0.286	2	0.05
	d					0.066	0.525	0.602
	dur					0.084	0.58	0.564
**Perform**		0.04	-0.01	0.798	0.5			
	const					0.000	0.000	1
	num					0.158	1.062	0.293
	d					0.011	0.084	0.833
	dur					0.069	0.458	0.649

Note: freq: eye-contact periods frequency; num: eye-contact periods total number; d: average child gaze distance d; dur; average eye-contact periods duration. GQ: Global Developmental Quotient; SA: Social Abilities subscale.

*p<0.05; **p<0.01; ***p<0.001

Table S3. Sub-groups pairwise comparisons.

**Sub-groups**		**Mean diff.**	***SE***	**t**	***p***
**0 vs 1**					
Eye-contact	num	-31.667	4.287	-7.387	***
	freq	-0.577	0.059	-9.615	***
	dur	-0.25	0.074	-3.36	**
	d	7.837	97.858	0.08	0.9
Clinical	ADOS	2.561	1.044	2.452	*
	Coordination	-12.582	4.951	-2.541	*
	Social	-11.153	6.362	-1.753	0.195
	Perform	1.823	6.691	0.272	0.9
	Age	-5.944	3.15	-1.887	0.152
**0 vs 2**					
Eye-contact	num	-2.625	4.624	-0.568	0.82
	freq	-0.024	0.065	-0.376	0.9
	dur	-0.077	0.08	-0.965	0.595
	d	-340.542	105.548	-3.226	**
Clinical	ADOS	0.171	1.126	0.152	0.9
	Coordination	4.859	5.34	0.91	0.626
	Social	5.921	6.862	0.863	0.653
	Perform	17.245	7.217	2.39	0.052
	Age	-22.962	3.397	-6.759	***
**1 vs 2**					
Eye-contact	num	29.042	4.713	6.162	***
	freq	0.552	0.066	8.377	***
	dur	0.173	0.082	2.11	0.097
	d	-348.379	107.591	-3.238	**
Clinical	ADOS	-2.39	1.148	-2.082	0.103
	Coordination	17.441	5.443	3.204	**
	Social	17.074	6.994	2.441	*
	Perform	15.423	7.357	2.096	0.1
	Age	-17.018	3.463	-4.914	***

Note: freq: eye-contact periods frequency; num: eye-contact periods total number; d: average child gaze distance d; dur; average eye-contact periods duration.
*p<0.05; **p<0.01; ***p<0.001
